# Literary Notes

**Published:** 1899-07

**Authors:** 


### LITERARY NOTES.

The American Medical Quarterly is the title of a new magazine that made its appearance in June. It is edited by Dr. William Warren Potter, of Buffalo, and is published in New York, by the American Medical Quarterly Company, 503 Fifth Avenue. It contains original articles by a number of the most prominent writers in the United States and Europe and is a high-class magazine in every respect.

On remittance to us of $3.00, subscribers will receive for one year The Buffalo Medical Journal and the American Medical Quarterly. If any physician does not find each issue of the latter publication worth more than the cost of the yearly subscription, the publishers offer to refund the money. They claim that no medical periodical in this country or Europe excels it in the character and value of the matter it contains.

---

The Buffalo State Hospital has issued its twenty-seventh annual report to the state commission in lunacy. It shows an expenditure of $220,998.82 foi the fiscal year ending September 30, 1897. During this time the daily average of patients under care was 1,193. This is one of the largest hospitals for the insane in the world and its management, business, administrative and professional, is economic, able and skilful.

---

The Buffalo General Hospital has published its fortieth annual report, which is for the year 1898. It is a well-printed brochure of about 100 pages containing finance, business and professional items of interest. It sets forth the work of the year in good form and makes a creditable showing for the hospital which has recently been much enlarged to meet the ever growing demands upon its space.

---

The Buffalo Hospital of the Sisters of Charity has just issued a souvenir commemorative of its fiftieth anniversary. It is a beautiful brochure of forty pages, profusely illustrated and printed on heavy book paper. The cover, in royal purple, is illuminated in gold. This interesting historical sketch is edited by Dr. Byron H. Daggett, of Buffalo, one of the special attending surgeons to the hospital. It is on sale at the hospital; price, 25 cents.

---

The *Iris*, Volume II., for the year 1899, edited and published by the undergraduates of the University of Buffalo, has just been issued. It is a quarto volume of near 300 pages, printed on heavy plate paper, copiously illustrated with etchings, drawings and half-tones. It is printed and bound by the Peter Paul Book Company. The work reflects great credit upon its editorial staff.

---

The Arlington Chemical Company, of Yonkers, recently purchased Mr. W. Granville Smith's painting entitled, The Country Doctor. It is a canvas, four feet by six, which has won distinction and place for the painter, as one of the coming men among the younger American artists. This picture was exhibited at the Columbus meeting of the American Medical Association, where it elicited great admiration.

